# Endoscopic ultrasound fine-needle biopsy vs fine-needle aspiration for lymph nodes tissue acquisition: a systematic review and meta-analysis

**DOI:** 10.1093/gastro/goac062

**Published:** 2022-11-03

**Authors:** Antonio Facciorusso, Stefano Francesco Crinò, Paraskevas Gkolfakis, Daryl Ramai, Andrea Lisotti, Ioannis S Papanikolaou, Benedetto Mangiavillano, Ilaria Tarantino, Andrea Anderloni, Carlo Fabbri, Konstantinos Triantafyllou, Pietro Fusaroli

**Affiliations:** Gastroenterology Unit, Department of Surgical and Medical Sciences, University of Foggia, Foggia, Italy; Department of Medicine, Gastroenterology and Digestive Endoscopy Unit, The Pancreas Institute, University Hospital of Verona, Verona, Italy; Department of Medicine, Gastroenterology and Digestive Endoscopy Unit, The Pancreas Institute, University Hospital of Verona, Verona, Italy; Department of Gastroenterology, Hepatopancreatology, and Digestive Oncology, CUB Erasme Hospital, Université Libre de Bruxelles (ULB), Brussels, Belgium; Gastroenterology and Hepatology, University of Utah Health, Salt Lake City, UT, USA; Gastroenterology Unit, Hospital of Imola, University of Bologna, Imola, Italy; Hepatogastroenterology Unit, Second Department of Internal Medicine-Propaedeutic, Medical School, National and Kapodistrian University of Athens, ‘Attikon’ University General Hospital, Athens, Greece; Gastrointestinal Endoscopy Unit, Humanitas Mater Domini, Castellanza, Varese, Italy; Endoscopy Service, Department of Diagnostic and Therapeutic Services, IRCCS-ISMETT, Palermo, Italy; Digestive Endoscopy Unit, Humanitas Clinical and Research Center—IRCCS, Rozzano, Milano, Italy; Gastroenterology and Digestive Endoscopy Unit, Forlì-Cesena Hospitals, AUSL Romagna, Forlì-Cesena, Italy; Hepatogastroenterology Unit, Second Department of Internal Medicine-Propaedeutic, Medical School, National and Kapodistrian University of Athens, ‘Attikon’ University General Hospital, Athens, Greece; Gastroenterology Unit, Hospital of Imola, University of Bologna, Imola, Italy

**Keywords:** EUS, FNA, FNB, cancer, metastases, lymphoma

## Abstract

**Background:**

Endoscopic ultrasound (EUS)-guided tissue acquisition represents the choice of methods for suspected lymph nodes (LNs) located next to the gastrointestinal tract. This study aimed to compare the pooled diagnostic performance of EUS-guided fine-needle biopsy (EUS-FNB) and fine-needle aspiration (EUS-FNA) for LNs sampling.

**Methods:**

We searched PubMed/MedLine and Embase databases through August 2021. Primary outcome was diagnostic accuracy; secondary outcomes were sensitivity, specificity, sample adequacy, optimal histological core procurement, number of passes, and adverse events. We performed a pairwise meta-analysis using a random-effects model. The results are presented as odds ratio (OR) or mean difference along with 95% confidence interval (CI).

**Results:**

We identified nine studies (1,276 patients) in this meta-analysis. Among these patients, 66.4% were male; the median age was 67 years. Diagnostic accuracy was not significantly different between the two approaches (OR, 1.31; 95% CI, 0.81–2.10; *P *=* *0.270). The accuracy of EUS-FNB was significantly higher when being performed with newer end-cutting needles (OR, 1.87; 95% CI, 1.17–3.00; *P *=* *0.009) and in abdominal LNs (OR, 2.48; 95% CI, 1.52–4.05; *P *<* *0.001) than that of EUS-FNA. No difference in terms of sample adequacy was observed between the two approaches (OR, 1.40; 95% CI, 0.46–4.26; *P *=* *0.550); however, histological core procurement and diagnostic sensitivity with EUS-FNB were significantly higher than those with EUS-FNA (OR, 6.15; 95% CI, 1.51–25.07; *P *=* *0.010 and OR, 1.87; 95% CI, 1.27–2.74, *P *=* *0.001). The number of needle passes needed was significantly lower in the EUS-FNB group than in the EUS-FNA group (mean difference, −0.54; 95% CI, −0.97 to −0.12; *P *=* *0.010).

**Conclusions:**

EUS-FNA and EUS-FNB perform similarly in LN sampling; however, FNB performed with end-cutting needles outperformed FNA in terms of diagnostic accuracy.

## Introduction

Lymphadenopathy represents a diagnostic challenge for clinicians. Detecting lymph node (LN) involvement from a neoplastic disease with the ability to distinguish metastases from benign or inflammatory conditions plays a fundamental role in tumour staging and treatment [1].

Therefore, imaging-guided LN sampling is commonly required to ascertain the underlying diagnosis and to assess adequate clinical management and patient prognosis. Among the available techniques, endoscopic ultrasound (EUS)-guided sampling is a valuable tool in the diagnostic management of thoracic and abdominal LNs, and is currently preferred over more invasive techniques such as mediastinoscopy and laparotomy [2].

Lesion diameter of >10 mm, hypoechogenic pattern, distinct edges, and round shape represent the main EUS characteristics of malignant LNs; high tissue stiffness at EUS-elastography and inhomogeneous arterial enhancement or pathological washout on contrast-enhanced harmonic EUS constitute additional malignant features [3–6]. Unfortunately, even when detecting these characteristics, simple morphology assessment is not sufficient to reliably differentiate benign from malignant LNs, hence highlighting the need for proper tissue sampling for a pathological confirmation of the underlying aetiology [7, 8].

It is well known that the diagnostic accuracy of EUS-guided fine-needle aspiration (EUS-FNA) for LNs is inferior to that for solid tumours of abdominal organs [9–12]. In the last few years, the widespread use of newer fine-needle biopsy (FNB), characterized by an end-cutting design, has allowed a theoretically higher ability to capture core tissues than traditional needles. However, there is still limited evidence on the comparison between EUS-FNA and EUS-FNB in LN sampling. Therefore, the aim of our meta-analysis was to compare the diagnostic outcomes and safety profile of EUS-FNB and EUS-FNA in patients with lymphadenopathy.

## Materials and methods

### Selection criteria

Studies included in this meta-analysis were randomized–controlled trials (RCTs) or retrospective comparative series that met the following inclusion criteria: (i) patients: adult patients with mediastinal or abdominal lymphodenopathy of unclear origin; (ii) intervention: EUS-guided LN tissue sampling through FNB (reverse-bevel [ProCore^®^, Cook Medical Inc., Bloomington, Indiana, USA], Franseen needle [Acquire^®^, Boston Scientific, Marlborough, Massachusetts, USA], and the Fork-tip needle [SharkCore^®^, Medtronic, Dublin, Ireland]); (iii) comparator: EUS-FNA of LNs; and (iv) outcomes: primary outcome was diagnostic accuracy, and secondary outcomes were histological core procurement, sample adequacy, diagnostic sensitivity, specificity, number of needle passes. Safety data were also analysed.

We excluded (i) non-comparative single cohort studies, (ii) case series with <10 patients per arm, (iii) studies not reporting any of the aforementioned outcomes, and (iv) studies evaluating endobronchial ultrasound-guided sampling of mediastinal LNs.

### Search strategy

Computerized bibliographic search was performed on PubMed/MedLine and Embase with no language restriction through August 2021, independently by two authors (A.F., P.G.) using the following search string with MeSH terms: ‘endoscopic ultrasound’ OR ‘eus’ AND ‘lymph node’ OR ‘lymphadenopathy’ AND ‘biopsy’ OR ‘aspiration’.

A complementary manual search was performed on additional databases (Google Scholar, Cochrane library) and by checking the references of all the main review articles on this topic, in order to identify possible additional studies. In cases of overlap publications from the same population, only the most recent and complete articles were included.

The quality of the included studies was assessed by two authors independently (A.F., S.F.C.) according to the Cochrane Collaboration’s tool for assessing the risk of bias for RCTs and the Newcastle-Ottawa scale for non-randomized studies [13, 14]. Any disagreements were addressed by re-evaluation and following a third opinion (P.F.).

### Outcomes

Primary outcomes were as follows: (i) diagnostic accuracy, defined as the summary of true positives (TPs) + true negatives (TNs) on the total number of patients. Gold standard for diagnosis was considered surgery or the evolution of the disease assessed for ≥6 months by a combination of clinical course and/or imaging studies [15]; (ii) diagnostic sensitivity, computed as the proportion of positives correctly identified with the test (TPs) on the prevalence of disease in the study cohort (TPs + false negatives [FNs]); and (iii) diagnostic specificity, calculated as the proportion of negatives correctly identified as such (TNs) among the patients who were not affected by the disease in the study cohort (TNs + false positives [FPs]). Additional outcomes were (i) sample adequacy, defined as the proportion of samples that were adequate for diagnosis; (ii) optimal histologic core procurement, defined as the proportion of patients with samples adequate for histological diagnosis; (iii) number of needle passes needed to obtain adequate samples; and (iv) adverse event rate.

### Statistical analysis

Study outcomes were pooled and compared between the two groups through a random-effects model based on the DerSimonian and Laird test, and results are expressed in terms of odds ratio (OR) or mean difference and 95% confidence interval (CI), when appropriate [16].

Presence of heterogeneity was calculated through *I*² tests with *I*² of <30% interpreted as low-level heterogeneity and *I*^2^ between 30% and 60% as moderate heterogeneity [17]. Any potential publication bias was verified through visual assessment of funnel plots.

Sensitivity analyses in the context of the primary outcome were based on study design (RCT vs retrospective), FNB needle used (end-cutting vs reverse-bevel), availability of rapid on-site evaluation (ROSE) (yes vs no), and location of sampled LNs (abdominal vs mediastinal).

All statistical analyses were conducted using RevMan version 5 from the Cochrane Collaboration. For all calculations, a two-tailed *P*-value of <0.05 was considered statistically significant.

## Results

### Included studies

**Figure 1.**
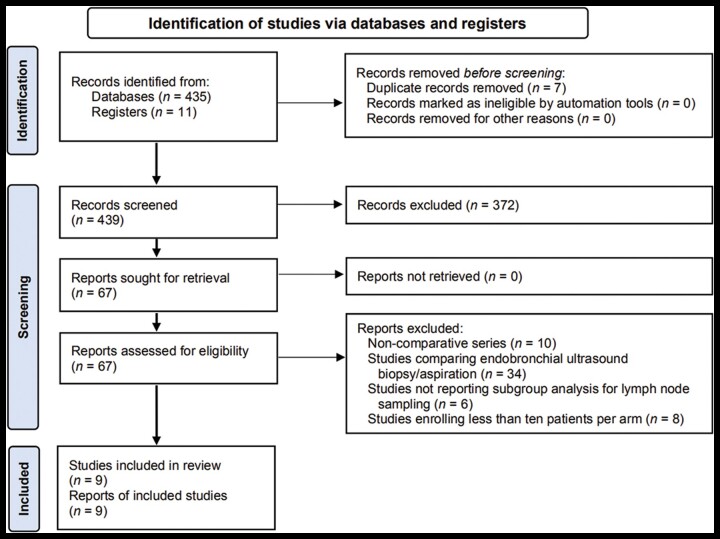
Flow chart of the included studies

From 446 studies identified using the search strategy, we included 9 studies [18–26] (Figure 1), recruiting 1,276 patients, with a median age of 67 years. The recruitment period ranged from 2011 to 2021. Out of these nine studies, five were retrospective series [18–20, 25, 26] and four were RCTs [21–24], of which three had a cross-over design (i.e. with the same patient undergoing both interventions in a randomized order) [21–23].

Main baseline characteristics of the included studies are summarized in Table 1. Four studies were conducted in patients with different lesions; only the data concerning LN sampling were considered in this meta-analysis [18, 22–24]. Baseline patient- and lesion-related characteristics were well balanced between EUS-FNA and EUS-FNB groups, with males comprising the majority of participants (66.4%) in the included studies. The mean lesion size ranged from 16 to 40 mm and most of the sampled LNs were abdominal. Particularly, two studies recruited exclusively patients with abdominal LNs [23, 26], whereas two other studies enrolled >90% of patients with abdominal location of the sampled LNs [20, 25]. Slow pull was used only in one study [18] and the fanning technique in four studies [18, 20, 21, 26]. ROSE was available for the majority of patients in three studies [18, 21, 23].

**Table 1.** Characteristics of the nine studies included this meta-analysis

Study	Country	Study period, design	Treatment	Age	Percentage of males	Lesion size (mm)	Location abdominal, diagnosis of malignancy	Fanning	Suction/slow pull	Needle	ROSE
Bang 2019^a^ [18]	USA	2014–2017	EUS-FNB: 88	67.1 ± 12.9	54.4%	28.8 ± 13.2	NR, NR	Yes	Slow pull	22G SharkCore^®^ or Acquire^®^	Yes
		Retrospective	EUS-FNA: 264	65.8 ± 13.7	56.7%	26.9 ± 13.9				22G or 25G FNA	
Chin 2017 [19]	Spain	2012–2015	EUS-FNB: 105	Overall: 63.8 ± 15.0	Overall: 117 (69.6%)	Overall: 20.3 ± 9.9	48 (45.7%)	NR	Suction	19G, 20G, 22G, 25G ProCore^®^	No
		Retrospective	EUS-FNA: 63				30 (47.6%)				
										19G, 22G, 25G FNA	
De Moura 2020 [20]	USA	2016–2019	EUS-FNB: 101	62.9 ± 13.2	61 (60.4%)	17.2 ± 8.7	95 (94%)	Yes	NR	20G, 22G, 25G SharkCore^®^, Acquire^®^,ProCore^®^	18 (17.8%)
							30 (35.3%)				
		Retrospective	EUS-FNA: 108	64.4 ± 11.7	81 (75%)	16 ± 7.3					
							89 (82.4%)			22G, 25G FNA	18 (16.6%)
							24 (30.4%)				
Hedenström 2021 [21]	Sweden	2014–2019	EUS-FNB: 48	Overall:	Overall:	Overall:	Overall:	Yes	Suction	22G ProCore^®^	38 (79.1%)
										25G FNA	
							17 (36%)				
		RCT cross-over	EUS-FNA: 48	69 (59–76)	24 (50%)	40 (30–51)	28 (58%)				
Hucl 2013^a^ [22]	India	2011–2012	EUS-FNB: 76	Overall:	Overall:	Overall:	Overall:	NR	Suction	22G ProCore^®^	No
										22G FNA	
							12 (15.7%)				
		RCT cross-over	EUS-FNA: 76	45.1 ± 14.2	43 (56.5%)	36.1 ± 12.3					
							13 (17.1%)				
Nagula 2018 [23]^a^	USA	2012–2014	EUS-FNB: 18	67.8 ± 12.7	53.2%	21.9 ± 8.1	100%/NR	NR	Suction for FNA	22G or 25G ProCore^®^	82%
										22G or 25G FNA	
		RCT parallel	EUS-FNA: 28	65.2 ± 13.2	51.1%	23.9 ± 16	100%/NR				
Sterlacci 2016 [24]^a^	Germany	2011–2013	EUS-FNB: 13	Overall:	51.8%	NR	NR	NR	Suction	22G ProCore^®^	No
		RCT cross-over	EUS-FNA: 13	68 ± 12						22G FNA	
							7 (53.8%)				
Tanisaka 2021 [25]	Japan	2013–2020	EUS-FNB: 71	70 (61–74)	36 (50.7%)	26 (19–35)	65 (91.5%)	NR	Suction	Acquire^®^	No
							89.6%			FNA	
		Retrospective	EUS-FNA: 83	67 (60–74)	51 (61.4%)	22 (15–35)					
							77 (92.8%)				
Facciorusso 2021 [26]	Italy, India, Colombia, USA	2012–2021	EUS-FNB: 105	64.4 ± 7.0	67 (63.8%)	21.4 ± 2.1	100%	Yes	NR	22G or 25G SharkCore^®^, Acquire^®^, ProCore^®^	No
										22G or 25G FNA	
							94 (89.4%)				
		Retrospective propensity matched	EUS-FNA: 105	64.6 ± 5.0	68 (64.7%)	22.4 ± 1.8					
							100%				
							85 (80.8%)				

Data are reported as absolute numbers (percentages) or mean (± standard deviation or with range).

a Only data concerning lymph node sampling were considered.

EUS, endoscopic ultrasound; FNA, fine-needle aspiration; FNB, fine-needle biopsy; RCT, randomized–controlled trial; ROSE, rapid on-site cytologic evaluation; NR, not reported.

Two studies used exclusively newer end-cutting FNB needles (SharkCore^®^ or Acquire^®^) [18, 25], five studies exclusively used a reverse-bevel FNB needle (ProCore^®^) [19, 21–24], whereas the remaining two studies used any of the aforementioned FNB devices [20, 26]. Four studies used 22G FNB needles [18, 21–23] while either 22G or 25G FNB needles were used in the other studies [19, 20, 24–26].

Final pathology on LNs assessment varies significantly among studies: benign LNs were found in 10.4%–51.8% of cases [19, 25], metastases from pancreatic cancer in 30.0%–71.3% [18, 19], haematological malignancies in 11.0%–33.0% [20, 25], and finally metastases from colorectal cancer were diagnosed in ≤70.6% in one study [26].

Quality assessment of the studies is summarized in Supplementary Table 1. Five studies were felt to be at low risk of bias [20–22, 25, 26], whereas four studies had higher risk of outcome reporting bias or selection bias [18, 19, 23, 24].

### Diagnostic accuracy

**Figure 2.**
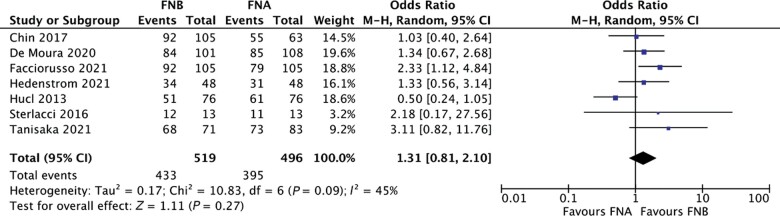
Forest plot of diagnostic accuracy analysis No significant difference between fine-needle biopsy (FNB) and fine-needle aspiration (FNA) was observed (odds ratio, 1.31; 95% confidence interval, 0.81%–2.10%; *P *=* *0.27). Evidence of moderate heterogeneity (*I*^2^* *=* *45%) was registered.

Overall, based on seven studies [19–22, 24–26] (519 patients in the EUS-FNB and 496 in the EUS-FNA group), pooled accuracy was 84.2% (95% CI, 77.1%–91.3%) and 80.4% (95% CI, 75.0%–85.9%) in the FNB and FNA groups, respectively. There was no significant difference between the two approaches (OR, 1.31; 95% CI, 0.81–2.10; *P *=* *0.270). Evidence of moderate heterogeneity (*I*^2^* *=* *45%; Figure 2) and no publication bias were found (Supplementary Figure 1a).

As presented in Table 2, sensitivity analyses restricted to study design and availability of ROSE confirmed the results of the main analysis, mainly with low to moderate heterogeneity. On the other hand, FNB performed with newer end-cutting needles clearly outperformed FNA (pooled accuracy with newer FNB needles, 89.2% [95% CI, 81.6%–96.8%]; OR, 1.87 [95% CI, 1.17–3.00]; *P *=* *0.009) with no evidence of heterogeneity (*I*^2^* *=* *0%) whereas no difference was observed in the comparison between reverse-bevel FNB and FNA (pooled accuracy with reverse-bevel needle, 81.2% [95% CI, 77.5%–91.5%]; OR, 1.03 [95% CI, 0.51–1.51]; *P *=* *0.630, *I*^2^* *=* *19%). FNB showed significantly higher accuracy than FNA also in the subgroup of abdominal lesions (OR, 2.48; 95% CI, 1.52–4.05; *P *<* *0.001) with no evidence of heterogeneity (*I*^2^* *=* *0%), whereas no difference was observed in the subgroup of mediastinal LNs (OR, 0.99; 95% CI, 0.36–2.73; *P *=* *0.980) although this finding should be interpreted with caution due to the low number of studies.

**Table 2.** Sensitivity analysis concerning diagnostic accuracy

Variable	Subgroup	No. of studies	No. of patients	Odds ratio (95% CI)	Within-group heterogeneity (*I*^2^)
Study design	RCT	3	EUS-FNB: 137	1.27 (0.38–1.97)	13%
			EUS-FNA: 137	*P *=* *0.740	
	Retrospective	4	EUS-FNB: 382	1.66 (0.89–2.54)	1%
			EUS-FNA: 359	*P *=* *0.120	
FNB needle	End-cutting needles	3	EUS-FNB : 277	1.87 (1.17–3)	0%
			EUS-FNA: 296	*P* 0.009	
	Reverse-bevel	4	EUS-FNB: 242	1.03 (0.51–1.51)	19%
			EUS-FNA: 200	*P *=* *0.630	
Availability of ROSE	Yes	2	EUS-FNB: 149	1.29 (0.24–2.57)	28%
			EUS-FNA: 56	*P *=* *0.690	
	No	7	EUS-FNB: 519	1.45 (0.85–2.47)	49%
			EUS-FNA: 440	*P *=* *0.170	
Location	Abdominal	4	EUS-FNB: 284	2.48 (1.52–4.05)	0%
			EUS-FNA: 290	*P *<* *0.001	
	Mediastinal	2	EUS-FNB: 38	0.99 (0.36–2.73)	0%
			EUS-FNA: 51	*P *=* *0.980	

CI, confidence interval; EUS, endoscopic ultrasound; FNA, fine-needle aspiration; FNB, fine-needle biopsy; RCT, randomized–controlled trial; ROSE, rapid on-site cytologic evaluation.

### Secondary outcomes

As presented in Table 3, based on six studies [18, 22–26], no difference in terms of sample adequacy was observed between the two treatments (OR, 1.40; 95% CI, 0.46–4.26; *P *=* *0.550, *I*^2^* *=* *40%). Again, no evidence of publication bias was observed (Supplementary Figure 1b). Pooled adequacy was 96.5% (95% CI, 93.4%–99.6%) with FNB and 95.3% (95% CI, 92.2%–98.5%) with FNA.

**Table 3.** Meta-analysis results for secondary outcomes of the study

Outcome	No. of studies	No. of patients	Odds ratio (95% CI)	Within-group heterogeneity (*I*^2^)
Sample adequacy	6	EUS-FNB: 371	1.40 (0.46–4.26)	40%
		EUS-FNA: 569		
Histological core procurement	3	EUS-FNB: 294	6.15 (1.51–25.07)	36%
		EUS-FNA: 477		
Diagnostic sensitivity	5	EUS-FNB : 338	1.87 (1.27–2.74)	0%
		EUS-FNA: 357		
Diagnostic specificity	8	EUS-FNB: 537	1.90 (0.53–6.78)	36%
		EUS-FNA: 524		
Number of needle passes	7	EUS-FNB: 507	−0.54 (−0.97 to −0.12)^a^	35%
		EUS-FNA: 712		

a This value is mean difference followed by 95% confidence interval.

CI, confidence interval; No., number.

Based on three studies [18, 20, 26], histological core procurement was significantly superior with EUS-FNB (OR, 6.15; 95% CI, 1.51–25.07; *P *=* *0.010, *I*^2^* *=* *36%), with pooled rates of 92.4% (95% CI, 86.3%–98.5%) and 67.6% (95% CI, 53.3%–81.8%) in the EUS-FNB and EUS-FNA groups, respectively.

**Figure 3.**
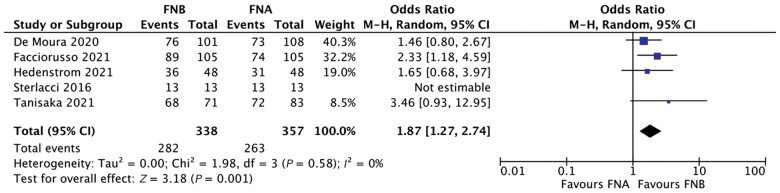
Forest plot of diagnostic sensitivity analysis Diagnostic sensitivity was significantly superior in the fine-needle biopsy (FNB) group as compared to fine-needle aspiration (FNA) (odds ratio, 1.87; 95% confidence interval, 1.27%–2.74%), with no evidence of heterogeneity (*I*^2^* *=* *0%).

As depicted in Figure 3, diagnostic sensitivity was significantly higher in the FNB group than in the FNA group (OR, 1.87; 95% CI, 1.27–2.74; *P *=* *0.001), with no evidence of heterogeneity (*I*^2^* *=* *0%). Specifically, pooled sensitivity was 85.9% (95% CI, 76.9%–95%) with FNB and 77.5% (95% CI, 65.9%–89.1%) with FNA.

Based on eight studies (all studies except Bang *et al*. [18]), pooled specificity was 98.8% (95% CI, 97.1%–100%) with FNB and 97.4% (95% CI, 95%–99.7%) with FNA, with no difference between the two approaches (OR, 1.90; 95% CI, 0.53–6.78; *P *=* *0.320, *I*^2^* *=* *36%).

The number of needle passes needed to obtain diagnostic samples was significantly lower in the FNB group than in the FNA group (mean difference −0.54; 95% CI, −0.97 to −0.12; *P *=* *0.010; *I*^2^* *=* *35%).

No procedure-related adverse events were observed in any studies included in the present meta-analysis.

## Discussion

Evidence on EUS-guided tissue acquisition in patients with abdominal lymphadenopathy remains scarce and conflicting, particularly concerning the comparison of EUS-FNB vs standard FNA. To the best of our knowledge, the current manuscript represents the first meta-analysis in the field and allowed us to make some key observations.

First, pooled accuracy with FNB was not statistically higher than that with FNA (84.2% vs 80.4%, *P *=* *0.270)—a result confirmed even in the absence of ROSE. However, when considering only the lesions biopsied with the newer end-cutting needles, FNB clearly outperformed FNA (pooled accuracy with newer FNB needles, 89.2%; OR, 1.87; 95% CI, 1.17–3.00) [27]. On the other hand, no significant difference was observed in the comparison between classical reverse-bevel FNB and FNA (pooled accuracy with reverse-bevel needle, 81.2%; OR, 1.03; 95% CI, 0.51–1.51). This aspect could be at least partially responsible for the results observed in previous comparative studies, using mainly a reverse-bevel device, that failed to show a superiority of FNB in LN sampling [19, 21, 24]. Therefore, whenever newer needles are available, FNB should be considered the first choice for tissue acquisition of LN tissue.

Moreover, as already observed in the aforementioned study by De Moura *et al*. [20], FNB performed significantly superiorly to FNA in the subgroup of abdominal lesions (OR, 2.48; 95% CI, 1.52–4.05), which represents the main field of interest for EUS-guided LN sampling in gastrointestinal endoscopy. However, no difference was observed in the subgroup of mediastinal lesions, although this finding should be interpreted with caution due to the limited number of studies.

Of note, while the results of the main analysis were characterized by moderate heterogeneity, sensitivity analysis showed a considerable decrease in heterogeneity (*I*^2^ of <20%), thus strengthening our confidence in the estimates obtained in this meta-analysis.

Our second observation is that no difference in terms of sample adequacy was observed between the two sampling methods (OR, 1.40; 95% CI, 0.46–4.26), with very high rates of adequate samples with both approaches (96.5% with FNB and 95.3% with FNA). On the other hand, as expected, histological core procurement with EUS-FNB was significantly superior to that with EUS-FNA (pooled rates, 92.4% vs 67.6%; OR, 6.15; 95% CI, 1.51–25.07). As already observed in other settings [18], FNB needles, in particular with the newer end-cutting design, are able to provide in a higher number of cases adequate samples for cell block analysis and histological evaluation, which is of paramount importance in several conditions such as lymphoma subclassification.

Third, diagnostic sensitivity was significantly higher in the FNB group than in the FNA group (OR, 1.87; 95% CI, 1.27–2.74), whereas pooled specificity was similar between the two strategies (98.8% vs 97.4%: OR, 1.90; 95% CI, 0.53–6.78). This finding confirms that the real limitation of tissue sampling with EUS-FNA is sensitivity, while false-positive rates (that characterize an impaired specificity) are usually uncommon even with standard fine-needle aspiration—again a finding consistent with other abdominal masses [28].

Fourth, as expected, the number of needle passes needed to obtain adequate diagnostic samples was significantly lower in the FNB group than in the FNA group (mean difference, −0.54; 95% CI, −0.97 to −0.12); this aspect represents a further advantage of FNB needles, as increasing the number of passes could result in a higher risk of adverse events and delayed procedural times. It could be also justified by the fact that during FNB, the specimen is visible and the endosonographer can judge it adequate or not for the final diagnosis by the macroscopic on-site evaluation. This technique showed high diagnostic yield and accuracy; moreover, the diagnostic performance further improved if tissue sampling was performed with large FNB needles and more than two passes [29].

Finally, no procedure-related adverse events were observed in any of the included studies, thus confirming that EUS-guided tissue acquisition is safe and can be routinely performed in clinical practice.

This study has some limitations. First, the number of included studies and recruited patients was relatively limited and the evidence was based both on retrospective series and RCTs. Furthermore, the included RCTs were unblinded, and hence prone to performance bias. It should be noted that this bias is not avoidable in endoscopy studies as the operator cannot be blinded to the device used. However, several sensitivity analyses were conducted in order to take into account all the potential confounders in the analysis. Moreover, the assessment of the risk of publication bias based on visual inspection of the funnel plots should be interpreted with caution due to the limited number of studies. Second, a subgroup analysis based on needle size could not be performed due to the lack of data. However, current evidence speaks in favour of comparability between 22G and 25G FNA and FNB [10, 30], hence it could be unlikely to find difference in this regard in the setting of LN sampling. Moreover, other relevant subgroup analyses based on technical aspects of tissue sampling such as the use of a fanning or suction technique could not be performed. However, most of the patients recruited in the included studies were sampled with the use of suction and thus our results should be considered applicable mainly to this strategy. Of note, the use of ROSE was not found to significantly influence the diagnostic performance with EUS-FNB in a recent large multicenter RCT [21] on solid pancreatic tumours. On the other hand, the presence of ROSE seems to increase the diagnostic yield of repeated EUS-FNA after previous non-diagnostic or inconclusive results [32]. In summary, this aspect was unlikely to impact the results of our analysis. Finally, another limitation is the fact that cost considerations were beyond the scope of the present study and could not be addressed; however, it is well known that FNB needles (especially the newer ones) are more expensive than needles used for FNA and this sometimes is a major contributing factor on what type of needle is finally used in various endoscopic units around the world, where the economic settings are not the same.

In conclusion, despite the aforementioned limitations, we think that our meta-analysis provides robust evidence on the comparison between EUS-FNB and EUS-FNA in patients with lymphadenopathy. Based on our findings and results, although EUS-FNB could not still be preferred to standard EUS-FNA, newer FNB needles deserve to be explored in further studies and, if their superiority over FNA is confirmed, they could be considered the diagnostic tool of choice in tissue sampling of LNs.

## Supplementary Data

Supplementary data is available at *Gastroenterology Report* online.

## Funding

The authors received no support or funding for this study.

## Conflict of Interest

The Authors declare no conflict of interest for this manuscript.

## Supplementary Material

goac062_Supplementary_Data
