# Supplementary material for: Endoscopic ultrasound fine-needle biopsy vs fine-needle aspiration for lymph nodes tissue acquisition: a systematic review and meta-analysis
Source: Gastroenterol Rep (Oxf). 2022 Nov 3;10:goac062. doi: 10.1093/gastro/goac062 (PMC9632631; doi:10.1093/gastro/goac062)
Supplement: goac062_Supplementary_Data [file goac062_supplementary_data.zip › 2022-255 Suppl. Table 1.docx]

**Supplementary Table 1.** Risk of bias assessment and quality of included studies

| Observational studies^a^ | | | | | | | | | | | | | |  |
| --- | --- | --- | --- | --- | --- | --- | --- | --- | --- | --- | --- | --- | --- | --- |
| Study | |  | | **Selection** | | | **Comparability** | | **Outcome** | | | **Overall quality** | |  |
| Bang 2019 | | | | * | | | ** | | * | | | L | |  |
| Chin 2017 | | | | ** | | | ** | | ** | | | M | |  |
| De Moura 2020 | | | | *** | | | ** | | ** | | | H | |  |
| Facciorusso 2021 | | | | *** | | | ** | | ** | | | H | |  |
| Tanisaka 2021 | | | | *** | | | ** | | ** | | | H | |  |
| Randomized controlled trials ^b^ | | | | | | | | | | | | | |  |
|  | **1** | | **2** | | **3** | **4** | | **5** | | **6** | **7** | |  | |
| Hedenstrom 2021 | L | | L | | H | L | | L | | L | L | | H | |
| Hucl 2013 | L | | L | | H | L | | L | | L | L | | H | |
| Nagula 2018 | L | | L | | H | L | | H | | L | L | | L | |
| Sterlacci 2016 | L | | L | | H | L | | H | | L | L | | L | |
| L, low; H, high; U, unclear; M, moderate.  ^a^ Study quality assessment performed by means of Newcastle/Ottawa scale (each asterisk represents if the respective criterion within the subsection was satisfied)  ^b^ Cochrane Collaboration’s tool for assessing the risk of bias across 7 domains: 1 (Random sequence generation), 2 (Allocation concealment), 3 (Blinding of participants and personnel), 4 (Blinding of outcome assessment), 5 (Incomplete outcome data), 6 (Selective reporting) and 7 (Other bias). | | | | | | | | | | | | | | |
