# Supplementary figures and images for: Endoscopic ultrasound fine-needle biopsy vs fine-needle aspiration for lymph nodes tissue acquisition: a systematic review and meta-analysis
Source: Gastroenterol Rep (Oxf). 2022 Nov 3;10:goac062. doi: 10.1093/gastro/goac062 (PMC9632631; doi:10.1093/gastro/goac062)

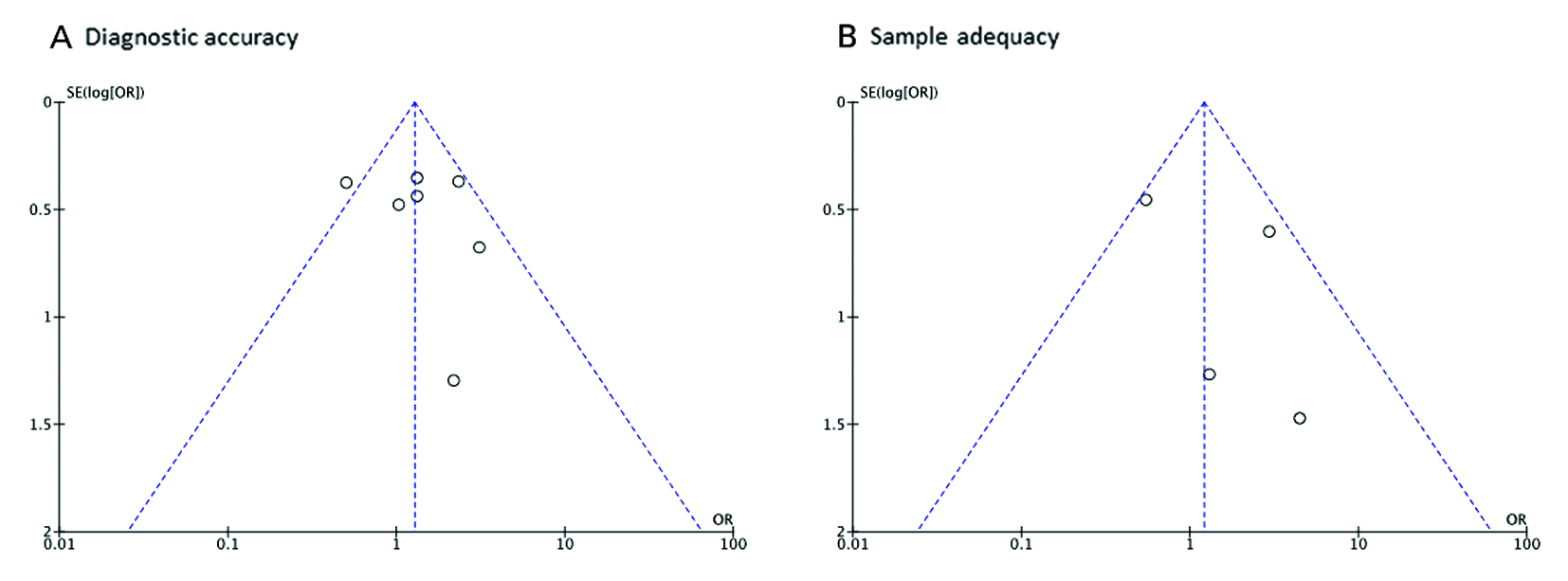

Supplement: goac062_Supplementary_Data [file goac062_supplementary_data.zip › 2022-255 Suppl._Figure_1.tif]
